# A Modified Behavior Risk Factor Surveillance System to Assess Diabetes Self-management Behaviors and Diabetes Care in Monterrey Mexico: A Cross-sectional Study

**DOI:** 10.3389/fpubh.2017.00097

**Published:** 2017-05-02

**Authors:** Marylyn Morris McEwen, Rogelio Andrès Elizondo-Pereo, Alice E. Pasvogel, Irene Meester, Javier Vargas-Villarreal, Francisco González-Salazar

**Affiliations:** ^1^College of Nursing, University of Arizona, Tucson, AZ, USA; ^2^Mel and Enid Zuckerman College of Public Health, University of Arizona, Tucson, AZ, USA; ^3^Basic Sciences Department, University of Monterrey, Monterrey, Mexico; ^4^Laboratory of Cellular Physiology, Northeast Center of Research, Mexican Institute of Social Security, Monterrey, Mexico

**Keywords:** type 2 diabetes, type 2 diabetes mellitus, HbA1c, behavioral risk factor surveillance system, diabetic complications, diabetic feet, diabetic retinopathy

## Abstract

Type 2 diabetes mellitus (T2DM) is one of the leading causes of death from worldwide non-communicable diseases. The prevalence of diabetes in the Mexico (MX)–United States border states exceeds the national rate in both countries. The economic burden of diabetes, due to decreased productivity, disability, and medical costs, is staggering and increases significantly when T2DM-related complications occur. The purpose of this study was to use a modified behavioral risk factor surveillance system (BRFSS) to describe the T2DM self-management behaviors, diabetes care, and health perception of a convenience sample of adults with T2DM in Monterrey, MX. This cross-sectional study design, with convenience sampling, was conducted with a convenience sample (*n* = 351) of adults in the metropolitan area of Monterrey, MX who self-reported a diagnosis of T2DM. Potential participants were recruited from local supermarkets. Twenty-six diabetes and health-related items were selected from the BRFSS and administered in face-to-face interviews by trained data collectors. Data analysis was conducted using descriptive statistics. The mean age was 47 years, and the mean length of time with T2DM was 12 years. The majority was taking oral medication and 34% required insulin. Daily self-monitoring of feet was performed by 56% of the participants; however, only 8.8% engaged in blood glucose self-monitoring. The mean number of health-care provider visits was 9.09 per year, and glycated hemoglobin level (HbA1c) was assessed 2.6 times per year. Finally, only 40.5% of the participants recalled having a dilated eye exam. We conclude the modified BRFSS survey administered in a face-to-face interview format is an appropriate tool for assessing engagement in T2DM self-management behaviors, diabetes care, and health perception. Extension of the use of this survey in a more rigorous design with a larger scale survey is encouraged.

## Introduction

Type 2 diabetes mellitus (T2DM) is a serious chronic disease and a major global health threat. T2DM is one of the leading causes of death from non-communicable diseases worldwide (1). In Mexico (MX), Barquera et al. (2) reported in 2013 that about 8 million people suffer from this disease, and it is the second leading cause of medical consultation for non-infectious disease. Other reports, published in 2010, report the prevalence of diabetes in MX ranges between 9.2 and 17% (3, 4). This rate compares to the MX–United States (U.S.) border states (Nuevo León, Tamaulipas, and Texas) that have an estimated T2DM prevalence of 17%, which is the highest T2DM rate at the national level in both countries (4). Monterrey, the capital of the state of Nuevo Leon and is the third largest metropolitan area in and the second wealthiest city in MX. Furthermore, Monterrey is considered the most Americanized city in the country (5). During 2012, the non-age-adjusted prevalence of T2DM was 15.5% (6) and 14.1% (7) in Nuevo Leon and Monterrey, respectively.

The economic burden of T2DM-related medical costs, disability, and decreased productivity is staggering; MX–U.S. estimated the nationwide T2DM-related direct and indirect costs amounted to USD$ 778 million in 2010 (2, 8, 9) and $245 billon USD, respectively (10). T2DM-related direct medical costs increased 14% during 2005–2010 due to complications (2).

In MX, several guidelines have been used for T2DM treatment and prevention of diabetes-related complications. The official norms, NOM-015-SSA2-2010 and NOM-015-SSA2-1994 (11, 12), contrast with guidelines operationalized in other health-care organizations and governmental institutions. For example, the Mexican Institute of Social Security (IMSS) and the Institute of Social Security and Services of State Workers (ISSSTE) have their own guidelines and protocols for the treatment and prevention of T2DM (13–16), which are different than the official norms. Mexican health-care providers who use these guidelines (17) provide routine health-care visits, diabetes education, and patient engagement in diabetes self-management behavior (11–17).

Mexico has not developed a national registry for diabetes. The prevalence of diabetes at the regional, national, and state levels is obtained from several national surveys that have recently been consolidated into the Mexican National Nutrition Survey (ENSANUT). As a result, the health statistical system in MX is considered high quality, primarily due to ENSANUT, which is collected every 6 years. MX recently initiated a medical specialties system and an information system for diabetes outcomes, especially related to quality of care indicators, is also planned. Analysis of the 2006 ENSANUT data demonstrated that adequate diabetes control is rare; HbA1c levels are infrequently evaluated, and, when evaluated, only 6.6% of the HbA1c measures were <7% (<53 mmol/mol) (2). Currently, there are no surveys collected in MX that assess T2DM daily self-management behaviors, diabetes care and health perception more frequently that every 6 years. A method for assessing these variables in the required timelines is required for T2DM self-management support and treatment decision support and for achieving optimal outcomes (17). The modified behavioral risk factor surveillance system (BRFSS) is proposed to address this gap.

The BRFSS is an ongoing, cross-sectional, multistage design survey (18, 19). The BRFSS is a telephone survey administered to the U.S. adult population to collect uniform state-specific data on preventive health practice and risk behaviors associated with chronic diseases, injuries, and preventable infectious diseases. The survey is composed of a set of core questions and modules (e.g., diabetes). The BRFSS is developed, coordinated, and funded by the Centers for Disease Control and Prevention (CDC). Details about the BRFSS design, purpose, sampling, validity, and reliability are available through the CDC website (18, 19). In 2011, BRFSS data collection, structure, and weighing methodology were revised to accommodate data collection by cellular telephones. An iterative proportional fitting, also known as raking, was applied to the BRFSS to improve the ability of a sample to reflect state level sociodemographics. The raking method was used to weigh the 2013 BRFSS data to increase the value of extremely low weights, decrease the value of extremely high weights, and reduce errors in outcome estimates (19). The BRFSS is not conducted in MX.

The purpose of this study was to use a modified BRFSS to describe the T2DM self-management behaviors, diabetes care, and health perception of a convenience sample of adults with T2DM in Monterrey, MX.

## Materials and Methods

### Study Design, Sample, Setting, and Data Collection

This cross-sectional study was conducted in the metropolitan area of Monterrey (Nuevo Leon, Mexico), a city located in the north of the country and 134 miles south of the MX–U.S. border, with an estimated population in 2015 of 4,406,054 habitants distributed in 12 municipalities. The study protocol was approved by Universidad de Monterrey, State health commission, institutional ethics, and research committees. Participants were recruited by trained data collectors at the main doors of local supermarkets before they entered the establishment. Inclusion criteria were (1) age: ≥18 years; (2) living in the metropolitan area of Monterrey, Nuevo Leon; (3) diagnosed with T2DM by a medical doctor. Women diagnosed with gestational diabetes were excluded from the study. There was no monetary incentive for participation in the survey. Potential participants were screened by the data collectors to determine if they met the inclusion criteria. Individuals who met the inclusion criteria and agreed to participate provided signed informed consent.

Cross-sectional data using a modified BRFSS in a convenience sample were collected at seven local supermarkets located in the main municipalities of the metropolitan area of Monterrey, Nuevo Leon (Monterrey, San Nicolas, San Pedro, Santa Catarina, Guadalupe, Apodaca, and Escobedo) between August and September 2015. Based on the populations’ distrust of telephone surveys, face-to-face interviews were hypothesized to be a more acceptable method for data collection. The majority of individuals recruited from the supermarkets agreed to participate in the face-to-face interviews. The interviews were conducted by three trained data collectors who were medical students recruited from the scholarship service program at the University of Monterrey. They were trained by a medical doctor familiar with the face-to-face interviewing format. The data collectors were oriented to the modified BRFSS survey and supervised in the administration of the survey until all questions were accurately administered. The data collector asked the questions in a semi-private location of the supermarket and entered the participant’s responses onto the survey form.

### Modified BRFSS Instrument

The modified BRFSS was composed of selected variables (*n* = 26) from the 2015 Spanish language BRFSS. All data were self-reported data. The 10 demographic variables included age, gender, marital status, education level, employment status, annual household income, body mass index (BMI) computed from self-reported height and weight, and exercise and smoking habits. The health-care access section included questions on health-care insurance and personal health-care provider. The 12 questions on T2DM health management behaviors and diabetes-related services collected information on the number of years with T2DM, age at diagnosis, type of T2DM medication (insulin or tablets), and the frequencies of blood sugar monitoring and feet check-ups, T2DM-related visits to health-care providers, professional check-up of HbA1c, feet sores, and dilated eye exams. With respect to T2DM education, the survey included questions to verify whether information on complications (sight, retinopathies, and nephropathies) had been provided and a course or class on diabetes self-management had been taken. The survey concluded with four questions used to describe the participants’ general physical and mental health perception during the last 30 days, and whether this affected daily activities.

### Statistical Analysis

Data obtained from participants were entered into an excel database and analyzed using the descriptive statistics version 22 of the Statistical Package in Social Science software (SPSS Inc., Chicago, IL, USA). The outcomes from qualitative variables were reported as percentages, while data from quantitative variables were reported as means and SDs.

## Results

Findings from cross-sectional data collected using the modified BRFSS in a convenience sample (*n* = 351) of adults residing in the metropolitan area of Monterrey, Nuevo Leon, MX are presented in Tables 1–3. Almost two-thirds (62.7%) were women and the mean age was 59.36 ± 11.5 years. Most of them were married, had a high school education or higher, and were not currently employed. More than half (76.9%) reported a monthly income less than $20,000 USD. Almost all (94.97%) reported having health-care insurance, primarily from IMSS and/or “seguro popular,” and 75.5% reported having a personal health-care provider. Self-reported weight and height data were used to calculate BMI; 74.1% of the participants had a BMI greater than 25.0. The majority (85.2%) were not current smokers and almost half (45.6%) reported they exercised. These data are reported in the Table 1. T2DM-related information, including T2DM self-management and professional T2DM-related care and education, is reported in Table 2. The mean age at which T2DM was diagnosed was 47 years and the mean time since T2DM diagnosis was 12 years. In terms of medication taken to manage T2DM, most (75%) took tablets to control T2DM, while only 34% used insulin. A daily check of feet was performed by 56%, but only 8.8% said they checked blood glucose level on a daily basis. On average, T2DM patients visited a health-care provider 9.09 (±6.8) times per year for T2DM control. HbA1c level was checked 2.6 (±2.7) times per year. Importantly, 40.5% reported never having a pupil dilation eye examination. With respect to general health perception, although most patients (79%) said they felt fair to good, about 8 (±10) days per month they felt physically and/or mentally bad, and about 4 (±8) days per month their lack of health interfered with daily activities (Table 3).

**Table 1:** **Demographic data**.

Characteristic	*N* = 351^a^
Gender	
Male	131 (37.3%)
Female	220 (62.7%)
Mean age (years, ±SD)	59.36 (11.5)
Marital status	
Married	258 (73.5%)
Not married	92 (26.2%)
Education completed	
≤High school	235 (67.0%)
≥High school	116 (33.0%)
Currently employed	
Yes	146 (41.6%)
No	205 (58.4%)
Income (USD)	
≤$20,000/year	270 (76.9%)
>$20,000/year	45 (12.8%)
Health-care insurance	
Yes	333 (94.9%)
No	16 (4.6%)
Personal health-care provider	
Yes	265 (75.5%)
No	86 (24.5%)
Body mass index	
≤25 kg/m^2^	58 (16.5%)
>25 kg/m^2^	260 (74.1%)
Exercise	
Yes	161 (45.6%)
No	188 (53.6%)
Current smoker	
Yes	48 (13.7%)
No	299 (85.2%)

*^a^Proportions calculated counting only the responders*.

**Table 2:** **Diabetes care**.

Variable	*N* = 351
Age at diabetes diagnosis (years; mean, ±SD)	47.31 (12.3)
Duration diabetes (years; mean, ±SD)	12.26 (9.8)
Currently taking diabetes medication	272 (75.1%)
Currently taking insulin	120 (34.2%)
Frequency blood sugar check	
Never	79 (22.5%)
Daily	31 (8.8%)
Weekly	60 (17.1%)
Monthly	86 (24.5%)
Yearly	73 (20.8%)
Frequency sore feet check	
Never	53 (15.1%)
Daily	199 (56.7%)
Weekly	66 (18.8%)
Monthly	13 (3.7%)
Yearly	7 (2.0%)
Frequency T2DM-related HCP visit in past 12 months (mean, ±SD)	9.09 (6.8)
Frequency HbA1c checked by HCP in past 12 months (mean, ±SD)	2.61 (2.7)
Frequency of a professional feet check-up in past 12 months (mean, ±SD)	3.54 (4.7)
Eye exam with pupil dilation	
Never	142 (40.5%)
Within past month	29 (8.3%)
Within past year	90 (25.6%)
Within past 2 years	23 (6.6%)
2 or more years ago	56 (16.0%)
Informed that sight problems and retinopathy were T2DM complications	
Yes	45 (41.3%)
No	198 (56.4%)
Taken a T2DM management course or class	
Yes	147 (41.9%)
No	199 (56.7%)

*HbA1c, glycated hemoglobin level; T2DM, type 2 diabetes mellitus; HCP, health-care provider*.

**Table 3:** **Health perceptions**.

Health perception	*N* = 351
General health	
Excellent	20 (5.7%)
Very good	30 (8.5%)
Good	109 (31.1%)
Fair	168 (47.9%)
Poor	22 (6.3%)
Bad physical health (days/last month^a^; mean, ±SD)	7.97 (10.1)
Bad mental health (days/last month^a^; mean, ±SD)	8.14 (10.4)
Inability to perform daily activities due to poor mental or physical health (days/last month^a^; mean, ±SD)	4.15 (8.5)

*^a^Month was considered as 30 days*.

We do not have the exact proportion of response but the most common reason for which people refused to participate in this study was the lack of time (no showed data).

## Discussion

In this cross-sectional study, a modified BRFSS is composed of 26 items was used to examine the demographic characteristics, T2DM self-management care, professional health care, and general health perception among adults with T2DM residing in the metropolitan area of Monterrey, the most Americanized city in MX. Although our findings do not represent the Mexican population as a whole, they can be compared with previously reported studies.

The demographic characteristics of this Monterrey metropolitan cohort was older and had a higher level of education when compared to the Mexican cohort of the 2002 MX–U.S. border states diabetes study (18). Clearly, the metropolitan area of Monterrey is not representative of the country or the MX–U.S. border region; Monterrey is an urban community with several state and private universities and a higher socioeconomic level than other border communities. However, in this cohort, the average annual household income was ≤USD $20,000. The unemployment rate (34–39%) on the Mexican side was higher than in the 2002 survey (18). This may be due to the BRFSS limited item response options (employed or unemployed), while the 2002 survey was stratified for employed, student, retired, or working at home. Education and income operate in an inverse relationship with diabetes, the higher the education and income levels, the lower the rate of diabetes (20). The BRFSS data indicate that the T2DM prevalence (19.5%) among those with an annual income ≤USD$15,000 was twice the rate of those with an income ≥$50,000 (8.3%) (20). Socioeconomic factors, including income, are adversely related to incidence, prevalence, and health status. These outcomes are similar to those reported by the Pan American Health Organization (21) on the MX–U.S. border states.

Despite higher poverty and unemployment rates than previous border studies, the majority (94.9%) had health insurance and 75.5% reported having a personal health-care provider. Health-care access was slightly better in our study than the data reported in the ENSANUT 2006 T2DM care study (74.4%) (2). The difference may be due to either the different population or sociodemographic characteristics of the metropolitan area of Monterrey or the *Seguro Popular*, a type of medical insurance granted by the government in 2003 to protect the uninsured. *Seguro Popular* has increased the national health coverage for more than 50 million Mexican citizens (22) but does not offer a personal health-care provider. This may explain the finding that the health insurance coverage is greater than the percentage of patients with a personal health-care provider.

These cross-sectional data may reflect a selection bias. That is, those individuals who self-selected into this study may not share characteristics of the general population with T2DM. For example, potential participants whose T2DM is poorly controlled may be home-bound and/or unable to travel to the public places where the study was conducted.

Both the American Diabetes Association with the Standards of Medical Care (17) and the Mexican NOM (11) share recommendations regarding BMI (<25 kg/m^2^) and the importance of physical activity for T2DM patients. In our cohort, 74% of respondents had self-reported BMI values outside of optimal range (>25 kg/m^2^). Based on the majority of participants who self-reported, they exercised and recognizing that BMI is directly influenced by the balance between caloric intake and physical activity, these were not the expected BMI results. Of note is the proportion who self-reported they exercised is greater than other border studies (4, 23, 24). The MX–U.S. border region is an obesogenic region (4). Environmental and personal obesogenic factors include lack of time, physical pain, depression, being overweight, unsafe neighborhoods, and lack of exercising facilities (4, 24–27), a lower socioeconomic class with poor availability to high-quality foods (4) but easy access to low-priced, well marketed, high-calorie, and high-fat processed foods (28).

Despite almost complete coverage of health insurance, access to a personal health-care provider for the majority, and an average of 9.09 T2DM control visits per year, almost half (48.9%) of the participants reported not having their HbA1c level checked by a health care professional. An HbA1c level <7.0% (<53 mmol/mol) is associated with a lower rate of both T2DM-related microvascular complications, such as retinopathy and long-term macrovascular complications (8). Therefore, an HbA1c level <7.0% (<53 mmol/mol) is an important surrogate marker to prevent complications. According to the 2012 ENSANUT, only 11.2% of the Mexican population has their HbA1c level checked annually (3). Unfortunately, we do not know the average HbA1c values of our sample. The average frequency of HbA1c monitoring was reported to be twice a year. However, it is possible that respondents confused blood glucose with HbA1c; if so, monitoring of HbA1c may be even less frequent (28). Indeed, it has been reported that Mexican diagnosed with T2DM who have a low education level are not familiar with the HbA1c test and tend to confuse it with the glycemia blood test (28).

Another important measure to prevent complications is an annual comprehensive foot examination in all patients with T2DM (17). In our cohort, feet were examined by a physician about three times per year, which complies with the guidelines. However, BRFSS does not verify the specific steps of a foot exam, which should include inspection, assessment of foot pulses, and testing for loss of protective sensation (8). As reported in U.S. studies and should be carefully considered for Mexican citizens, foot-related complications among T2DM patients continue to increase despite evidence-based research documenting the effectiveness of comprehensive T2DM foot exams. It is essential that comprehensive foot exams be conducted by a health-care provider to reduce foot-related complications including amputations (29, 30).

Only one-fourth (25.6%) of the participants reported receiving a dilated eye exam in the last year. Interestingly, 40.1% of the cohort reported being told T2DM affected eyes or they had retinopathy. The frequency of eye exams for diabetes-related retinopathy reported in this study is consistent with findings in other border studies (31). Despite a high prevalence of retinopathy in diabetic patients in MX, preventive examination is not commonly conducted (32). The ADA recommends that individuals with T2DM should have an initial dilated and comprehensive eye examination, carried out by an ophthalmologist or optometrist, following T2DM diagnosis (17). If there is no evidence of retinopathy for one or more eye exams, then a schedule of every 2 years may be considered. However, if diabetic retinopathy is present, subsequent examinations should occur annually. Initial and subsequent eye examinations as recommended are critical to prevent blindness caused by diabetic retinopathy (17).

The ADA in the U.S. (17) and the MX NOM (11) provide evidenced-based guidelines for health-care professionals who provide T2DM care. However, the major health-care systems in MX (IMSS, ISSSTE, and *Seguro Popular*) and some medical associations (Federación Mexicana de Diabetes, Asociación Mexicana de Diabetes) follow different T2DM guidelines. The NOM recommends that patients with T2DM have annual HbA1c exams and, if visual alterations occur, T2DM patients should be referred to an eye care specialist. However, whether it is a lack of health-care professional referrals or a delay in care seeking by the patient, it is common that patients are not examined until they have severe ocular damage. A scenario that contributes to high retinopathy rates in MX (32).

The main limitations of the study are those of a self-reported sample survey and the method of data collection and potential selection bias related to the convenience sample. In the absence of a BRFSS infrastructure in MX, the Monterrey MX cohort data were collected using select items from the BRFSS administered to the U.S. population. However, we used face-to-face interviews and a different, less structured convenience sample. It is important to note that telephone surveys are not feasible in the metropolitan area of Monterrey as most people refuse to speak with persons who are unknown to them for safety reasons. The convenience sample, although collected from seven cities in the metropolitan area, may not have been representative of the larger population. Standardized protocols for data collection, including training of study personnel, were used to minimize interviewer bias between data collectors in the face-to-face interviews. Finally, the absence of data to report the numbers of individuals at each stage of the study (e.g., numbers screened for eligibility and numbers eligible) is another limitation.

Though the health literacy level was not assessed, it is possible, considering the education level, that participants did not understand all questions (28, 33). Furthermore, recall bias and social desirability may have contributed to measurement error. Other potential limitations include systematic error and non-response or refusal to participate, which may have possibly affected internal validity. Finally, diabetes self-management is complex.

Despite robust evidence for treatment protocols and self-management education for T2DM control and prevention of complications, a gap continues to exist in translating the evidence into clinical practice and engagement of residents on either side of the MX–U.S. border in T2DM self-management care. Overweight, older patients with a sedentary lifestyle and limited engagement in T2DM self-management behaviors, and health-care providers who do not follow the guidelines for medical care of patients with T2DM increases the probability of future T2DM-related complications.

The modified BRFSS was uniquely utilized to describe the T2DM self-management behaviors, diabetes care, and health perception of a convenience sample of adults with T2DM in Monterrey, MX. The use of this survey could be extended in a more rigorously designed, larger scale. For example, for binational data collection from Hispanic-Americans and Mexicans with T2DM living in the MX–U.S. border region. The recommendations put forth by de Cosío and colleagues (34) provide direction for the design and implementation of a binational survey and should be followed. Data collected using the modified BRFSS could be used to inform the development, effective targeting, and evaluation of future binational health T2DM interventions. Further testing of the modified BRFSS should be conducted.

## Ethics Statement

All subjects signed written informed consent. The protocol was approved by the “Ethic and Research Committee of University of Monterrey and State Health Commission.”

## Author Contributions

Write protocol: FG-S, JV-V, and IM. Train interviewers: FG-S and RE-P. Supervise logistic interview: MM, FG-S, and RE-P. Capture data and perform excel: RE-P. Perform analysis: MM and AP. Interpret outcomes: MM, AP, JV-V, and FG-S. Write manuscript, edit manuscript, and review final version: MM, IM, JV-V, and FG-S. Manuscript translation and English review: MM and IM.

## Conflict of Interest Statement

The authors declare that the research was conducted in the absence of any commercial or financial relationships that could be construed as a potential conflict of interest.
